# Providers, Unmarried Young Women, and Post‐Abortion Care in Kenya

**DOI:** 10.1111/sifp.12035

**Published:** 2017-09-22

**Authors:** Chimaraoke O. Izugbara, Carolyne P. Egesa, Caroline W. Kabiru, Estelle M. Sidze

## Abstract

Young women and girls in Kenya face challenges in access to abortion care services. Using in‐depth and focus group interviews, we explored providers’ constructions of these challenges. In general, providers considered abortion to be commonplace in Kenya; reported being regularly approached to offer abortion‐related care and services; and articulated the structural, contextual, and personal challenges they faced in serving young post‐abortion care (PAC) patients. They also considered induced abortion among young unmarried girls to be especially objectionable; stressed premarital fertility and out‐of‐union sexual activity among unmarried young girls as transgressive of respectable femininity and proper adolescence; blamed young women and girls for the challenges they reported in obtaining PAC services; and linked these challenges to young women's efforts to conceal their failures related to gender and adolescence, exemplified by pre‐marital pregnancy and abortion. This study shows how providers’ distinctive emphasis that young abortion care‐seekers are to blame for their own difficulties in accessing PAC may add to the ongoing crisis of post‐abortion care for young women and adolescent girls in Kenya.

Globally, adolescents disproportionately experience the adverse effects of unsafe abortion. About 60 percent of unsafe abortions in Africa occur among young women and girls under age 25 (World Health Organization 2011). In Kenya, young women and girls comprised about half of the patients treated for complications of unsafe abortions in 2012 (APHRC et al. 2013). In the same year and compared to their older counterparts, young abortion patients also experienced more severe complications of induced abortion (such as sepsis, shock, or organ failure); presented more often with repeat abortions; were less likely to receive contraceptives and counseling upon discharge; and tended to be managed with lower‐quality abortion care procedures such as dilation and curettage (D&C), forceps, and digital (finger‐only) evacuation (APHRC et al. 2013; Mutua, Maina et al. 2015; Ziraba, Izugbara et al. 2015).

Health providers are key to facilitating young post‐abortion patients’ access to quality abortion care, including appropriate clinical management, non‐stigmatizing treatment during care, and comprehensive contraceptive counseling (Harris, Debbink et al. 2011; Healy 2013; Mercier, Buchbinder et al. 2015). Current efforts to improve sexual and reproductive health equality have emphasized the need for providers who understand the particular issues young women face in relation to abortion, have non‐judgmental attitudes toward them, and can offer them quality abortion services, including post‐abortion contraception (Mayi‐Tsonga, Assoumou et al. 2012; Evens, Otieno‐Masaba et al. 2014; Mutua, Maina et al. 2015).

However, reports of providers’ violation and mistreatment of young abortion care‐seekers remain widespread (Dressler, Maughn et al. 2013; Ndunyu 2013; Izugbara, Egesa, and Okelo 2015). In Kenya, abortion care providers reportedly continue to act and relate inhospitably to young abortion patients, deliberately manage abortion patients without pain killers, abuse them sexually, and sometimes threaten to have the police arrest them (Center for Reproductive Rights 2010; Ndunyu 2013; Izugbara, Egesa, and Okelo 2015). In other instances, structural factors, including lack of training in post‐abortion care (PAC), lack of PAC equipment, and legal barriers, impede providers’ ability to effectively serve abortion patients (APHRC et al. 2013, 2015; Payne, Debbink et al. 2013). Within this context, doubts have been expressed, not only about providers’ appreciation of the challenges young women face in relation to abortion services but also about their capacity to offer empathic and quality unprejudiced care to young abortion patients (Steele and Chiarotti 2004; Evens, Otieno‐Masaba et al. 2014; Izugbara, Egesa, and Okelo 2015).

Notwithstanding their critical role in ensuring quality abortion care, little research has explored PAC providers’ understanding of the challenges young post‐abortion care patients face in seeking, reaching, and receiving treatment, particularly in contexts of high abortion stigma. This study presents findings from this key population to complement global knowledge on challenges of offering and receiving post‐abortion care in contexts of high abortion stigma. A focus on providers’ relationships with young abortion patients is key to understanding their challenges in serving patients; plugging gaps and disparities in reproductive health outcomes among young people; furnishing evidence for youth sexual and reproductive health (SRH) policy formulation and implementation; and informing strategies for addressing barriers to abortion patients’ access to respectful services worldwide.

In this article, we explore, *inter alia*, abortion care providers’ constructions of the challenges that unmarried young women and girls face in relation to abortion care‐seeking. We specifically ask: How do post‐abortion care providers in Kenya perceive young women and girls in relation to abortion? And what do they consider the challenges young women experience in obtaining PAC? We define young women and girls as those aged less than 25 years.

## CONTEXT

Kenya, with a revised Constitution that unambiguously addresses abortion, offers an ideal setting for interrogating the challenges associated with abortion‐related care in a developing country. The Constitution, which came into force in 2010, permits abortion when, in the opinion of a trained health professional, a woman's life or health is in danger. It also clearly allows trained health professionals, particularly medical doctors, gynecologists and obstetricians, and experienced midwives, to offer abortion. Critics, however, argue that while the Constitution notionally expands the grounds for legal abortion in Kenya, it does not fundamentally improve access to safe abortion services (Center for Reproductive Rights 2010; East Africa Center for Law and Justice 2012; APHRC et al. 2013). In addition to the absence of a definitive interpretation of the Constitution's stance on the legality of abortion, popular opinion in Kenya still holds abortion to be criminal and immoral. Arrests, harassment, and penalization of health providers suspected of offering safe abortion service also continue against a background of an unresolved lawsuit instituted by a consortium of human rights groups and activists in Kenya against the country's Ministry of Health over the non‐release of official guidelines for offering abortion care (Marlow, Wamugi et al. 2014; Izugbara, Egesa, and Okelo 2015).

Public attitudes toward abortion remain largely negative in Kenya. The majority of women and girls seeking abortion therefore usually obtain it clandestinely and unsafely, often with negative sequelae (East Africa Center for Law and Justice 2012; APHRC et al. 2015; Ziraba, Izugbara et al. 2015). Evidence shows a continuing trend among Kenyan women and girls to obtain abortions outside formal facilities or with the support of unqualified providers, and to present at formal facilities for the management of the consequent complications (Evens, Otieno‐Masaba et al. 2014; Izugbara, Egesa, and Okelo 2015). Available data indicate that the induced abortion ratio in Kenya is 30 abortions per 100 live births (APHRC et al. 2013).

### Literature and Theory

Research on abortion care providers has largely focused on their role in promoting positive care experiences and health among abortion patients. The World Health Organization placed responsibility for the safety of abortion in the hands of providers, defining abortion safety primarily in terms of the termination of a pregnancy by providers with the requisite skills (World Health Organization 2011). In many countries, abortion remains illegal, forcing many women to resort to unqualified abortion providers and settings, often with far‐reaching negative health sequelae (Francome 2015). The different types of unqualified providers who have emerged to fill the gaps created by the illegality and stigma of abortion are also amply documented (Jewkes, Gumede et al. 2005; Rasch 2011; Payne, Debbink et al. 2013).

Research showing that safe abortions—those performed by trained providers in hygienic settings—carry few health risks has inspired calls for the expansion of the number of safe abortion providers (Warriner, Wang et al. 2011; Healy 2013; Weitz, Taylor et al. 2013). In many countries, mid‐level providers, nurses, physician assistants, clinical officers, midwives, and community health workers have been trained to offer safe abortion services, with little or no difference in the quality and outcomes of abortion care offered by them and doctors (Warriner, Wang et al. 2011; Basnett, Shrestha et al. 2012; Samandari, Wolf et al. 2012; Healy 2013; Evens, Otieno‐Masaba et al. 2014; Puri, Tamang et al. 2015; Tamang, Puri et al. 2015). Research showing that training and supporting mid‐level health providers to offer abortion services can save women's lives and reduce health system costs has reinforced calls for task‐shifting in the context of abortion care (APHRC et al. 2015; Prada, Bankole et al. 2015).

The challenges facing providers of abortion care have emerged as a critical research theme. Studies show that, even in countries where abortion is legal, providers must deal with stigma, lack of equipment, poor training and logistical support, high demand for services, and professional isolation. Victimization, harassment, picketing, vandalism, and murder have been reported as other hazards that providers encounter (Harris, Martin et al. 2013; Forrest and Henshaw 1987; Harris, Debbink et al. 2011; Joffe 2014; Martin, Debbink et al. 2014). Studies on the attitudes and practices of providers toward abortion care‐seekers frequently show that providers can be both supportive and abusive toward care‐seekers (Steele and Chiarotti 2004; Mayi‐Tsonga, Assoumou et al. 2012; Evens, Otieno‐Masaba et al. 2014). The motivations and disincentives for offering abortion services among providers in different contexts have also been extensively documented (Gogna, Romero et al. 2002; Rance 2005; Okonofua, Hammed et al. 2011). Notwithstanding the extensive body of research on abortion care providers, it is largely silent on several relevant issues, particularly on how providers construct and understand the challenges abortion patients face in relation to care‐seeking. We address this knowledge gap using interview data from abortion care providers in Kenya.

The theory that abortion stigma derives largely from social roles and the constructions of gender has steered much of the current scholarship on attitudes and stigma toward women seeking and providers offering abortion services (Lipp 2011; Cockrill and Nack 2013; Abrams 2015). Building on Goffman's (1963) work, scholars argue that stigmatization related to abortion is a contextual and dynamic social process. Stigma against women who have had abortions tends to derive from a belief that abortion violates the ideals of femininity and womanhood. Kumar, Hessini, and Mitchell (2009) argue that abortion violates two fundamental ideals of womanhood: nurturing motherhood and sexual purity. The desire to be a mother is considered central to being a “good woman,” and non‐procreative sex is often considered illicit for women. Individuals stigmatized through close association with abortion—such as staff of health facilities that provide abortion; supporters of women who have had abortions; partners, family, and friends; and abortion researchers and advocates—are discredited because they are seen as contributing to the violation of female ideals of sexuality and motherhood. Abortion‐related stigma is further strengthened by anti‐abortion laws and beliefs that attribute personhood to the fetus and consider abortion to be dirty, unhealthy, unfeminine, and inhuman. However, the gendered stigma theory largely ignores the situation of unmarried young abortion‐seekers. It ignores the critical question of adolescence and its construction in relation to abortion stigma, raising the need for an expanded view on abortion stigma toward young unmarried women.

For Link and Phelan (2006), stigma is socially produced and driven by the contexts in which people and communities operate. In Kenya, evidence indicates that adolescence is constructed in terms of sexual abstinence, innocence, and restraint (Mitchell, Halpern et al. 2006, Marlow, Wamugi et al. 2013); that the majority of health providers lack focused training on non‐judgmental interaction and provision of adolescent‐responsive SRH service, including PAC and contraceptive counseling (Blommaert 2010; Kabiru, Izugbara, and Beguy 2013); that popular beliefs attribute personhood to the fetus and support notions that abortion is un‐womanly; and that there is growing public and media discourse about the deterioration of sexual and other virtues among young people in the country. Against this backdrop, we hypothesize that the labeling and stigmatization of young post‐abortion care‐seekers may be rooted in widespread cultural expectations that young people, in particular, should be incapable of objectionable acts such as terminating life, which is how abortion is frequently depicted in Kenya (Obengo 2013). In the current study, we postulate that Kenyan providers’ understanding of the challenges young women and girls face in relation to PAC will reflect these normative and widespread conceptions about proper femininity and adolescence in relation to abortion. These conceptions, we argue, will stress the innocence expected of young people; abortion as a violation of the ideals of femininity, adolescence, and womanhood; and female adolescents’ involvement in abortion as symbolic of cultural and moral deterioration.

## MATERIALS AND METHOD

### Sampling

Data for this study were collected from 152 PAC providers drawn from a sample of public and private facilities that offer PAC services in Kenya. These facilities were purposively selected because they (a) provide post‐abortion care and (b) treat large numbers of young PAC‐seekers based on data from a 2012 study on the incidence and magnitude of unsafe abortion in Kenya (APHRC et al. 2013). Although the sampled facilities may not be statistically representative of all health facilities in Kenya, they were selected from all regions and facility levels. Respondents were the primary providers of PAC services in the sampled facilities and were identified by the heads of the participating facilities. In total, 51 in‐depth interviews (IDIs) and ten focus group discussions (FGDs) were held. Verbal informed consent was obtained from all interviewees for their participation and for the audio‐recording of their responses. The characteristics of responding providers and their facilities are given in Table 1.

**Table 1:** Characteristics of participants in IDI and FGD

	**IDI (N=51)**	**FGD (N=101)**
**Characteristics**	**Number**	**Number**
**Sex**		
Male	30	65
Female	21	36
**Age**		
30 and below	12	19
31–40	19	45
41 and above	20	37
**Educational level**		
Secondary	1	0
College	42	94
University	8	7
**Profession**		
Nurse	33	78
Clinical Officer	11	22
Doctor/Gynecologist	7	1
**Years of experience**		
0–5	12	68
6–10	22	25
Above 10	17	8
**Facility level** a		
6	1	1
5	9	6
4	19	63
3	10	17
2	12	14
**Type of facility**		
Private	16	26
Public	35	75

a A health care facility level is a description of functionality as defined by the Kenyan Ministry of Health. Level 1 is the lowest level of health care, Level 6 is the highest.

### Data Sources

Data were collected using in‐depth individual qualitative interviewing and focus group discussion tools. The tools were informed by current literature and evidence regarding the challenges faced by abortion care providers and patients in East Africa (Nalwadda, Mirembe et al. 2011; Paul, Gemzell‐Danielsson et al. 2014; Izugbara, Egesa, and Okelo 2015; Mutua, Maina et al. 2015). The tools sought data on the socio‐demography of the providers; their understanding of the magnitude of the problem of abortion, particularly among unmarried young people and girls; their opinions on the drivers of induced abortion among young women; their viewpoints on the key challenges they face in caring adequately for young abortion care‐seekers; their opinions of women and young girls seeking pregnancy termination and post‐abortion care; and their ideas of the challenges young unmarried women and girls face in seeking and obtaining PAC services. The tools were also piloted among a sample of PAC providers in facilities.

All interviews were administered in English by trained research officers. Interviews lasted an average of 50 minutes, were all audio‐recorded, and held in a private setting. The interviewers were two post‐graduate SRH researchers with extensive experience in qualitative interviewing. The study guide was developed, reviewed, and approved by an international team of experienced qualitative SRH and abortion researchers.

### Analysis

Interview data were transcribed by an experienced researcher and then compared to the taped interviews by one member of the research team. At first, the interview data were concurrently but independently coded by the lead author and an expert qualitative data coder. Following Izugbara and Egesa (2014), the authors and coder met afterward to review the coding outcomes, ensure intercoder concordance, and agree on a codebook that mirrored the thematic groupings of the interview questions and the key issues emerging from the data. Based on the jointly developed codebook, transcribed interviews were then finally coded with Nvivo. A qualitative inductive approach involving thematic assessment of the narratives was adopted to interpret the data. This approach promotes the detection of overriding themes in qualitative data and interpretation of the meanings and messages of themes through the continual investigation of narrative data for categories, linkages, and properties. The current study is framed around three major themes that emerged from our analysis of the narrative data: perceptions regarding demand for abortion and post‐abortion care among adolescents; notions of the challenges in serving young PAC patients; and views of the challenges facing young women and girls in relation to PAC. Verbatim quotes have been used in the analysis to focus attention on major responses and themes. Our interpretation of the data was also shared with some of the respondents to ensure it represented their views. The study was reviewed and approved by the Ethics Review Boards of the Kenya Medical Research Institute. The Ministry of Health, Kenya also endorsed the study.

## RESULTS

### Perceived Demand for and Magnitude of Abortion in Kenya

In both the FGDs and IDIs, providers generally considered abortion to be commonplace in Kenya. They were also frequently approached by women and girls seeking pregnancy termination services. Individuals looking for pregnancy termination services for their wives, friends, daughters, sisters, and relatives also contacted providers. Corroborating the regularity of the demand for pregnancy termination services, a provider observed:

Interviewer (I): So, women and girls come often to you seeking abortion?
Provider (P): Of course. Even before I started [this interview] with you, one person called me, they want abortion…
I: They want abortion?
P: Yes, very many come to us for it.

The reported regularity of these requests notwithstanding, most providers said they did not offer pregnancy termination services, because they considered abortion illegal, belonged to religious faiths that forbid abortion, lacked basic equipment for safe abortion in their health facility, feared that colleagues and supervisors would not approve, dreaded reprisals from the community, and lacked training in safe pregnancy termination. The few providers who admitted offering safe termination services often highlighted the distinctiveness of the circumstances under which they offered the services. One provider had offered the service to a young rape victim, another to an HIV‐positive couple, and a third to a young school‐going relative.

Claims that repeat abortion was commonplace in Kenya were frequent. Many providers had seen abortion patients return, within a few months or years of PAC treatment, with a new episode of unsafe abortion. A widely held view was that both young and adult women were at risk for repeat unsafe abortion. Knowledge of safe abortion methods such as manual vacuum aspiration (MVA) and medical abortifacients like Misoprostol was high among the providers. They were also aware of many unsafe abortion methods used in Kenya. Providers reported having treated patients who had abortions performed by chemist shop operators, itinerant medicine vendors, traditional healers, TBAs, teachers, boyfriends, and relatives. Many had also treated patients who terminated their pregnancies by pumping cold air through the cervix, inserting sharp objects through the vagina, drinking potash and herbal concoctions, and having someone step repeatedly on their abdomen. Providers also knew of local abortion folklore and practices, including that abortions could be induced by performing extreme physical activities; inserting herbs and sharp objects into the vagina; overdosing on certain anti‐malarial medicines, emergency contraceptives, analgesics, and anti‐bacterial drugs; and drinking concentrated tea leaves.

The conditions for obtaining lawful pregnancy termination in Kenya were considered stringent. Many private providers reportedly exploited the situation to charge patients exorbitant fees for abortion services. Poor women and girls bore the brunt of the inequities in access to safe abortion services. Unsafe abortion and its sequelae such as disability, long‐term facility stay, and death were commonest among marginalized women and girls. Well‐off women were said to obtain safe abortion services from qualified providers and health facilities.

While there was recognition that all women of reproductive age are potentially at risk of induced abortion, providers frequently considered unmarried young girls and women to be at elevated risk. The typical responses to IDI questions about women most likely to seek abortion included: “Majority are young people below 24 years,” “We even see girls below 16,” “It is often unmarried young girls below 25 years,” and “I would say 25 and less and they are usually unmarried.” FGDs routinely generated similar responses:

Interviewer: So tell me about the ages and other characteristics of women and girls who present most for post‐abortion care in your facility.
Respondent 1 (R1): Young women and girls …they are the ones. Sometimes they are as young as 13 years.
R2: In my experience, the younger generation… often unmarried…below 25 years are more and maybe because they fear the consequences …
R3: In my experience at my facility, it is the young people and they are often not married.
R4: Majority are young.… My facility is near colleges so most of the people are students.… You might get one or two married people…
R5: Like the one I treated last month, very young girl, about 13 years old. She secured [abortion] unsafely through a traditional birth attendant.…

### Providers’ Challenges in Serving Young PAC Patients

Providers of PAC services reportedly faced both structural and personal challenges. The commonly identified structural challenges included inadequate staffing, equipment, and supplies; legal restrictions; lack of clear national guidelines on abortion‐related care; and lack of training for providers on youth‐focused abortion care. Stigma was the foremost individual‐level challenge.

Interview data indicated that very few providers had received trained in offering sexual and reproductive health services to youth. The majority of PAC providers in Kenya were reportedly unfamiliar with youth‐focused SRH services, do not understand young people's SRH care‐seeking practices, are unable to introduce the topic of contraceptives to them, and frequently disrespected and stigmatized young abortion care‐seekers. Youth‐focused and youth‐friendly services were considered rare in Kenya. The majority of facilities in the country, particularly public ones, were said to be poorly equipped, do not take the confidentiality needs of young abortion care‐seekers into consideration, or do not have separate and dedicated rooms and staff for youth. In most facilities, as providers observed, young abortion patients are kept in the same rooms with adults, attended to in the open, and treated discourteously. One doctor noted:

Many providers have a [bad] attitude when they find a young patient coming in with bleeding or abortion complications. Few of us know how to deal with young people with abortion complications. We don't even have a special place for them in most facilities. The patient has to pass through the same process just like other adults too. She is left to suffer shame…. Some don't want to touch a young person who has an abortion or bleeding; they scold them, so that is a major challenge.

Poor staffing, shortage of PAC providers trained in youth‐sensitive service provision, and lack of requisite equipment, tools, medications, and supplies were other constraints facing PAC providers. Some health facilities also lacked rooms for family planning and contraceptive counseling; did not own essential PAC equipment (such as for MVA); and regularly experienced stock‐outs of basic medications and family planning products. The situation in one facility was described thus: “When a person who requires MVA comes, she …waits for a long time before she is attended to because we have only one person who is trained to do that. From there, they …wait till the following day… to be seen for family planning services. So it is a challenge for us…” Articulating the problem of inadequately qualified staff and how it impedes the work of PAC providers, one respondent observed:

Sometimes the provider on duty doesn't know how to use an MVA and the patient has to wait for another staff, and when that staff arrives, he or she is overworked and not able to do it well either. You come back again after two days [for additional treatment] because it was not done well. Sometimes, a gynecologist will be involved to [treat complications] afresh.

The legal ambiguity surrounding abortion in Kenya negatively affects the quality of PAC services. Kenya's abortion law was considered to be inexact, providing a basis for the harassment of providers by the public and government agencies. Some providers have had to obtain police and/or institutional approvals before treating young abortion patients, resulting in delays, aggravated complications, and sometimes fatalities. One provider who treated a young patient presenting with incomplete abortion related how, two days after the girl's discharge, her parents and a local chief were at the facility with the police to arrest her for criminal pregnancy termination. “Luckily, I had taken all her records and the medical officer [in my facility] defended me [confirming that the girl had presented with an incomplete abortion]. But I realized that I have to be careful if I don't want to go to jail,” she noted.

Stigma by the media, colleagues, and the public toward health providers was also a frequently reported challenge among providers. Providers reported being regularly called names (such as abortionists) and insulted for providing PAC services. Others have been accused of responsibility for the pregnancies of their young patients. Stigmatization not only prevented providers from conscientiously doing their jobs, it also reportedly obstructed them from treating abortion patients courteously. One respondent said, “If colleagues see you treat a young abortion patient with much care…, they would start rumoring that it is because you got her pregnant….” And another respondent reported:

PAC providers suffer from stigma. People say that our work is to provide young people abortions. But we are really the people who save the lives of girls who undergo unsafe abortion. A whole facility can even experience stigma, too. Even some providers will say, ‘‘that facility… the only thing they do is abortion for young people.” Even the public says, “No, that place is not good, their work is just abortions.” So, when a young person presents with complications, one is careful what to do.

There was also evidence that stigma prevented providers from “working in the MVA room” because “they fear that they will be labeled as abortionists.” Prompt and respectful treatment of PAC patients in such facilities was often delayed. One nurse noted that PAC patients in her facility sometimes have to “wait for someone ready to be called names to serve them.” Male providers were particularly stigmatized. Data suggested that hospital staff frequently suspect them of hiding under the cover of PAC to provide pregnancy termination services to young women and girls. One provider observed, “When I was in another hospital and even in this district hospital, when a young girl comes and asks for a particular male nurse, it raises eyebrows and people know it is an abortion case…even some providers will be asking why must it be a man, why not a woman? So it is like identifying with such a thing and it makes male providers careful…”

### Unmarried Young PAC‐Seekers’ Challenges

Providers acknowledged that unmarried young PAC‐seekers also faced critical challenges. They linked these challenges to their defiance of norms of sexual abstinence and propriety. While FGDs and IDIs indicated respondents’ relative tolerance of abortion among married adult women, a prevalent belief was that unmarried young people should be abstinent. Adult married women could, reportedly, consider abortion if they were widowed, lost their jobs, had many children, or experienced contraceptive failure. According to several of the providers, many young people were initiating sex very early. This was seen as a marker of their immorality as well as a cause of unintended pregnancy and abortion. In sum, providers thought that unmarried young women's involvement in and efforts to hide their pre‐marital sexual relationships were responsible for the challenges they faced in obtaining PAC services. “Young people should not be having sex. They should focus on their education. It is not abortion that is their problem. It is their immorality…,” noted one provider.

Shame was the foremost challenge reportedly facing unmarried young women seeking post‐abortion care. Providers blamed young women's efforts to cover up their guilt and feminine failure exemplified by pre‐marital pregnancy and induced abortion. “When we advise them to avoid sex until they get married…they refuse, but tomorrow you will still see them at the facility with street abortion,” noted a provider. Stressing a sentiment that was prevalent throughout the interviews, another provider thought that “the biggest barrier they face is shame and their naivety. Once they go into their immorality and the pregnancy comes, they try to abort and they cannot come to us when things go wrong because of the shame they have brought on themselves. They know you will ask …how a young person like them is into sex and learnt to kill.” Another participant offered, “Sometimes, you see they know they are too young to have sex… where did she get pregnant, these children, they fear and they try to hide. It is the shame.…” We were also told: ‘They know they have done something very bad. They have had sex and also killed… they have difficulties because they want to hide their bad behavior.” Among young women and girls, shame was also a common explanation for delays in seeking PAC. Respondents noted that when young girls try to hide their immorality and the resultant abortion, they delay care‐seeking, ultimately increasing their risks for severe morbidities and death. One respondent's view was:

They come here when they are almost dead…in very bad condition. We begin to manage them late and sometime they are already in bad state and cannot survive…. So these people are just left somewhere …there was a case I saw and a youth died because of sepsis and this girl stayed at home till the last minute. She came to the hospital at night, around past midnight and with the parent who didn't know. When they came, we found it was abortion and it was septic. She died within 15 minutes on arrival. The mother was so shocked and cried. She said, “this girl… she washed my clothes yesterday, with all this pain and she couldn't tell me.’’ But the truth is that the girl was ashamed and hiding her bad behavior.

Embarrassment arising from perceived immorality was another explanation for young people's resort to informal abortion providers. According to providers, when young people become pregnant, they resort to crude and extreme abortion methods or unqualified abortion providers. As one provider observed, “They know they cannot come to us because we will not help.… So, they go to bad people.… They are trying to hide that they have had sex and become pregnant when they should be reading their books.” Another respondent noted, “Then there was another one, the father was a pastor and the mother a housewife, and they did not even know.… The teachers brought her to the hospital…. It was an abortion. She was very young and we were asking ourselves ‘Isn't this one too young for sex?’ But she had started and that is what they try to hide from you…their bad character.”

Narratives underscored that young abortion patients presenting at facilities also tended to conceal their conditions out of disgrace. In the view of one respondent, “In most cases, they are not very open at the start to tell you how it happened.… They tell lies because they know you will be angry with them…and this prevents you from helping them well.” Providers have had to discover, sometimes quite late, that a young woman under their care had undergone unsafe abortion. Experience has taught some providers to conduct thorough examinations when attending to young unmarried girls. “If you don't check well, you will be treating fever and pain, and the girl will die of sepsis,” one respondent noted. Providers had cared for young girls at their facilities who complained of physical assault, headache, pains, malaria, or fever, only to find out later that they have had an unsafe abortion. “They will never tell you what they did because they know they have been wayward and are now pregnant. So you may concentrate on treating the wrong thing.” One provider made her point as follows:

Let me give a short story. There was a girl, a school girl; I don't know what information she got from her friends.… She had conceived and tried to hide it by abortion. She came to hospital and told us that she is having diarrhea. She was there for three days. I was not the one directly in charge of the patient. It was my colleague. So my colleague didn't go into details to know the cause of the abdominal pain and so she concentrated on the diarrhea. The patient died. Young girls are like that. Once they get pregnant from their bad behavior, they try to hide it and they will not tell you the truth when they are seeking treatment.

The illegality of abortion was also mentioned as a challenge that young people faced in seeking and obtaining PAC services. The law prohibits abortion in most cases, forcing many women and girls seeking PAC services in facilities “to rather die at home or tell lies about what they have really done to themselves.”

## DISCUSSION AND CONCLUSION

The role of post‐abortion care providers in protecting the health and wellbeing of abortion care‐seekers is well documented. However, abortion care providers face several challenges in their work. Many of these have been previously reported and include lack of equipment and training in youth‐friendly services (Kennedy, Bulu et al. 2013), heavy workload, stigmatization by community and colleagues, and the illegality of abortion in Kenya (Paul, Gemzell‐Danielsson et al. 2014). The perceived illegality of abortion in Kenya results in delays in treating young abortion patients. Before commencing treatment, providers spend time on requisite documentation to show that they had not engaged in pregnancy termination. This is not surprising in a context where harassment of health providers by police and others is on the rise (Center for Reproductive Rights 2010; Evens, Otieno‐Masaba et al. 2014; Izugbara, Egesa, and Okelo 2015).

The present study identified providers’ distinctive understanding of the challenges that young people face in obtaining PAC services. While providers emphasized how structural challenges and stigma impeded their provision of services to PAC patients, they largely blamed young abortion care‐seekers for their own difficulties in reaching and using PAC. Blaming the victim is a pervasive aspect of providing care and treatment for stigmatized conditions (McCarraher, Chen‐Mok et al. 2010; Evens, Otieno‐Masaba et al. 2014). Victim‐blaming diverts responsibility and culpability for people's difficult situations from social structural factors to their behaviors and cultural patterns (Ryan 1971). In the context of our study, providers held firmly to the claim that the challenges young people faced in reaching and using PAC are their own fault. Providers’ explanations echoed forms of gender orthodoxy that emphasized expectations of chastity and abstinence among unmarried young women. For them, the origin of unmarried young women's ordeals in the context of PAC services was their attempt to conceal their failures exemplified by pre‐marital sex, out‐of‐union pregnancy, and induced abortion.

Research suggests that providers sometimes stigmatize women seeking abortion‐related care. This stigma often derives from a belief that abortion violates feminine ideals of womanhood. In the case of unmarried young women and girls, provider stigma can further derive from expectations of sexual innocence and purity among adolescents. Young women who have had non‐procreative sex and are seeking to exert control over their own reproduction can be potentially seen by providers as deviating from norms of respectable adolescence and gender roles. Scholars (Goffman 1963; Kumar, Hessini, and Mitchell 2009; Harris, Debbink et al. 2011; Lipp 2011; Norris, Bessett et al. 2011; Cockrill and Nack 2013; Abrams 2015) posit that providers’ stigmatization of abortion‐care services for young people may not always be related to the act of aborting a fetus, but rather in their conceiving an unwanted pregnancy, engaging in sexual activity, and failing to meet expectations of gender and adolescence.

That providers blamed young abortion care‐seekers for their plight is a critical finding. Besides averting the gaze of providers from the structural challenges that young people face in relation to sexual and reproductive health matters and promoting society's double standards with regard to male and female gendered expectations and accepted behavior, such a practice can promote provider abuse and mistreatment of young people (Okonofua, Hammed et al. 2011; Payne, Debbink et al. 2013; Evens, Otieno‐Masaba et al. 2014). Research shows that victim‐blaming conceals the discrimination that marginalized groups face on a regular basis, and obscures from providers the societal pressures behind the choices young people make. Often, victim‐blaming is a leading cause of secondary victimization by providers in the form of inappropriate post‐abortion treatment, stigma, and disrespect. Recently, Izugbara, Egesa, and Okelo (2015) observed that the stigma and discrimination that abortion patients in Kenya face in society at large are regularly reproduced and reinforced in formal health care settings. They present hospital‐related folklore as well as data on abortion care‐seekers’ narratives of insidious provider and health facility mistreatment in the form of threats to hand them over to the police, management with crude evacuation methods and without pain medication, and needless disclosures of young people's circumstances to others.

In conclusion, as Kenya continues to maximize the full potential of abortion care providers (in preventing unsafe abortion, guaranteeing access to quality and respectful abortion care, preventing repeat abortion, and ensuring high‐quality contraceptive counseling for abortion patients), our study demonstrates the urgent need for providers to shift from understanding abortion as a consequence of young women's and girls’ moral decline to being able to view unsafe abortion as one of the consequences of the denial of women's human rights (Fathalla 2006). Kenya's effective implementation of the WHO‐approved comprehensive post‐abortion care (CPAC) model, to which the country is a signatory, will enhance the quality of PAC; improve family planning counseling; and reduce provider stigma toward care‐seekers, delays in receiving care, and mistreatment of abortion patients. When effectively implemented, CPAC builds provider and community support for abortion care‐seekers, facilitates the supervision of abortion care‐providers, reduces stigma related to abortion at all levels, and ensures abortion care‐seekers’ access to quality and life‐saving sexual and reproductive health interventions and programs, including contraceptive information and services (Corbett and Turner 2003). Health policymakers in Kenya also need to accelerate the release of the long‐awaited national guidelines on the prevention and management of unsafe abortion complications.

### Study Limitations

Despite the important evidence from this study, it has some limitations. The study relied heavily on data from nurses and clinical officers, who are more likely than doctors to hold conservative opinions about abortion and young people (Okonofua, Shittu et al. 2005; Abdi and Gebremariam 2011; Payne, Debbink et al. 2013). Data were also collected from a sample of PAC providers only, and the conclusions reached should only be cautiously extrapolated to all providers of health care services in Kenya. The study was also conducted at a time when there was considerable discussion about the illegality, dangers, and immorality of abortion as well as youth degeneracy in Kenyan media and in political and religious circles. Further, given the generally high levels of abortion stigma in Kenya, it is not clear whether respondents aimed to disassociate themselves from abortion and its practice in the country.
